# Patients’ experiences of support for learning to live with diabetes to promote health and well-being: A lifeworld phenomenological study

**DOI:** 10.3402/qhw.v11.31330

**Published:** 2016-08-17

**Authors:** Karin Johansson, Sofia Almerud Österberg, Janeth Leksell, Mia Berglund

**Affiliations:** 1 Department of Health and Care Sciences, Faculty of Health and Life Science, Linnaeus University, Växjö, Sweden; 2 Department of Administration, Kronoberg County Council, Växjö, Sweden; 3 Primary Care, Region Kronoberg County Council, Växjö, Sweden; 4 School of Health and Social Sciences, University Dalarna, Falun, Sweden; 5 Department of Medical Sciences, Uppsala University, Uppsala, Sweden; 6 School of Health and Education, University of Skövde, Skövde, Sweden

**Keywords:** Diabetes, health, lifeworld, phenomenology, reflection, support for learning, well-being

## Abstract

Learning to live with diabetes in such a way that the new conditions will be a normal and natural part of life imposes requirements on the person living with diabetes. Previous studies have shown that there is no clear picture of what and how the learning that would allow persons to incorporate the illness into their everyday life will be supported. The aim of this study is to describe the phenomenon of support for learning to live with diabetes to promote health and well-being, from the patient’s perspective. Data were collected by interviews with patients living with type 1 or type 2 diabetes. The interviews were analysed using a reflective lifeworld approach. The results show that reflection plays a central role for patients with diabetes in achieving a new understanding of the health process, and awareness of their own responsibility was found to be the key factor for such a reflection. The constituents are responsibility creating curiosity and willpower, openness enabling support, technology verifying bodily feelings, a permissive climate providing for participation and exchanging experiences with others. The study concludes that the challenge for caregivers is to create interactions in an open learning climate that initiates and supports reflection to promote health and well-being.

Diabetes is a long-term illness that significantly alters one's life. The experience of learning to live with diabetes has been shown to involve understanding and controlling the changing body, as well as protecting the body from damage in both the short and long terms. This learning has been described in earlier studies from a lifeworld perspective (Berglund & Källerwald, 2012; Johansson, Almerud-Österberg, Leksell, & Berglund, 2015; Kneck, Klang, & Fagerberg, 2011). The term “lifeworld” refers to the natural attitude through which a person approaches himself/herself, other persons, and the world (Husserl, 1907/1989). From the lifeworld perspective, the human body is understood as a lived body that is at the same time biologically thinking, feeling, and acting (Merleau-Ponty, 1945/2002). Learning from a lifeworld perspective means an altered understanding created through reflection and dialogue that involves the whole being of his/her context (Bengtsson, 2006; Berglund, 2014; Ekebergh, 2007). For present purposes, learning is understood as integrating the illness as a change in the lived body with a new understanding of one's self as a person with diabetes (cf. Johansson et al., 2015). Studies informed by this definition of learning have described the importance for diabetes care of interactions that enable sharing of patients’ personal understandings of living with the disease (Adolfsson, Smide, Rosenblad, & Wikblad, 2009; Boström, Isaksson, Lundman, Graneheim, & Hörnsten, 2014; Jutterstöm, 2013; Zoffman et al., 2016).

Supporting patients’ learning processes and incorporating the illness into their lives require knowledge of how patients should be educated and how carers can satisfy their need for learning (Friberg & Hansson-Scherman, 2005). The importance of understanding patients’ learning processes and the need for support has been insufficiently emphasized in the literature. An integrative review has identified a need to clarify the nature of patient education as the basis for developing supportive activities (Friberg, Granum, & Bergh, 2012). One potential problem is that patient education is often organized according to a preplanned programme that defines patient needs in terms of identification of carers, placing greater emphasis on the medical component of the illness than on its existential element (Adolfsson et al., 2009). Toombs (1993) describes the patient perspective as *illness* (an internal perspective) and the medical perspective as *disease* (an external perspective), which gives rise to differing expectations about what patients need to learn and cope with. This may also affect how health care providers think and act in patients’ learning.

Support in living with the illness is defined as social and professional support. Hupcey (1998) describes social support as both existential and physical, which is experienced as complex because several parameters are involved in producing the desired effect. Professional support is mediated by caregivers in their practice (Hupcey & Morse, 1997) and generally follows guidelines and policies; this support can be emotional, but it is not the same as social support. Although the patient is at a disadvantage and needs to be reassured, caregivers may not necessarily trust the patient. Effective support in reaching treatment goals has been shown in different ways. One way is to involve and help patients in setting individual goals (Adolfsson et al., 2009), and give opportunities for self-monitoring blood glucose (Durán et al., 2010). Another key factor is time with the caregiver (Norris, Lau, Smith, Schmild, & Engelgau, 2002) and meeting nurses with knowledge of diabetes and pedagogical training (Adolfsson et al., 2009; Swedish Council on Technology Assessment in Health Care [SBU], 2009). Timing is also important in matching resources because support at the wrong time or unwanted support may be negatively perceived (Hupcey, 1998). Berglund, Westin, Svanström, and Sundler (2012) found that patients feel distrusted and mistreated when their perspective on illness is not taken into account. This, according to the authors, constitutes a barrier to learning.

The aim of patient education is that patients should feel secure and develop good self-care as well as capability, in which knowledge, motivation, training, and support are all important elements (Hunt, 2013). Diabetes self-management is seen as an ongoing process of facilitating the knowledge, skills, and ability required for diabetes self-care (Haas et al., 2014). Berglund (2014) demonstrated the potential to support patients’ learning using a didactic model based on lifeworld theory, in which the learning persons are challenged to reflect and to personally decide how they wish to live with their illness. Learning to live with long-term illness is an existential issue to reduce stress and maintain and enhance short- and longer term health and well-being (Berglund, 2014). This focus on the patient's learning process raises the important question, “how learning to live with diabetes can be promoted?” The present study describes the phenomenon of support for learning to live with diabetes to promote health and well-being, from the patient's perspective.

## Methods

In this study, the phenomenon of support for learning to live with diabetes is explored and illuminated by the reflective lifeworld research (RLR) approach, based on phenomenological epistemology as described by Dahlberg, Dahlberg, and Nyström (2008).

### Participants and data collection

Following Dahlberg et al. (2008), interviews were used to explore patients’ experiences. Informants were recruited from four care units in South Sweden (one specialist clinic and three primary care units), using different forms of patient education. Each unit recruited three Swedish-speaking patients, varying in age, sex, duration of illness, and treatment. The informants were five men and seven women between 45 and 76 years of age, with illness duration ranging from 2 to 46 years. Three informants had type 1 diabetes and nine had type 2 diabetes; age of onset varied from 13 to 74 years. Informants chose the interview venue; five were conducted in the home and seven were conducted in the regular care unit. Interview duration varied between 45 and 75 min. The interviews were conducted in conversational form, beginning with an open question such as “how the patient experienced falling ill and how they learned to live with the illness?” Follow-up questions (e.g., *tell me more*, *in what way*, *how did you experience it*, and *what has been important for your learning*) were asked to gain deeper insight into the phenomenon.

### Data analysis

The method of analysis can be described as a dialectical process (Dahlberg et al., 2008), beginning with the whole, analysing its parts, and then reconstructing the whole to understand the essence of the phenomenon. Initial analysis of the text as a whole then turns to a focus on its parts to identify units of meaning: a word, a sentence, or a longer piece of text. These meanings are scrutinized against the background of the whole before building clusters or groups of related meanings. Following the analysis of units of meaning and clustering in groups, the next phase involves identifying the phenomenon's essence. According to Dahlberg and Dahlberg (2003), the essence can be understood as the core aspects of a phenomenon on an abstract level whereas the constituents describe the phenomenon on a concrete level. This can be understood as a new whole.

In this study, the analysis began by listening and reading through the interviews to become acquainted with their content before looking for similarities, differences, and patterns of meaning in the verbatim printed interviews. Questions were asked to the text about what was said, how it was said, and what is its likely meaning—for instance, how the informants described the experience of learning and what supported the learning process. By observing similarities and differences in the material, a pattern of experiences and meanings emerged, transforming the subjective lifeworld perspective expressed in the interviews into a professional and scientific description, focused on the studied phenomenon.

During the course of the research, patterns changed in character, requiring movement between the whole and parts before finally arriving at a description of the essence. The essential structure was further described in terms of its five constituents. In the results below, the essence is presented first, followed by its constituents and quotes to illuminate the findings.

### Ethical considerations

Approval for the study was granted by the Regional Ethics Committee of Linköping (Dnr 2012/222-32). Field officers approved the participation. Informants were provided with oral and written information about the aim of the study before giving written consent.

## Results

Learning to live with diabetes is supported by self-responsibility, driven by reflection on experiences, curiosity, and a desire to understand and influence one's daily life and illness processes. Beginning from responsiveness to experience-based feelings in the lived body, reflection supports the ongoing learning process to promote health and well-being. The technology for measuring one's own blood glucose level is a component of this special support, confirming the body's feelings and in some cases raising questions that promote the process of reflection. Openness enables an ability of learning support from family and friends, as well as from professional caregivers. Activation of reflection, participation in decision-making, and responsibility are the cornerstones for learning and for a supportive climate. When experiences are explicitly shared with others, progress is made, and lessons are learned from less successful attempts. The phenomenon under study is further enlightened by its five constituents: responsibility creating curiosity and willpower, openness enabling support, technology verifying bodily feelings, a permissive climate providing for participation and exchanging experiences with others.

### Responsibility creating curiosity and willpower

Learning is supported by the patients’ awareness of and willingness to take responsibility for their own health situation, as seen in the patients’ eagerness to learn, their curiosity, and various forms of knowledge seeking to improve health. Responsibility supports learning as the patients reflect over their experiences and use their knowledge to calculate the risks and benefits of planned actions and to make conscious choices. One informant described this as follows:

Had my parents not had heart attacks, and had I not read online that there is the risk of a heart attack, I don't know if I would have been so active; I was really scared, and I still am. I know that my erectile function works, so that is not a concern, but the heart thing is something that is always at the back of my mind. Had it not been like this in my family, I don't think I would have been so hard on myself, I'm not sure.

Responsibility to support learning in a way that promotes health and well-being is reflected in how patients set their own targets for treatment of their illness, and take responsibility for a life with the illness and its treatment. This, in turn, is supported by reflection of the advantages of this approach, in terms of reduced risk of complications and of future suffering. One informant described how his learning was supported by his willingness and effort to achieve blood sugar level goals like this:

When my blood sugar is at the level of a healthy person, I feel really well, so that's where I want to be. If it is a little higher, at 6–7 and starting to approach 7, I of course think that I will have to make sure that it goes down a little with an extra dose of insulin.

The pursuit to reach goals supports responsibility, which manifests itself in creativity, and in being critically reflective, and analytical. The goal is to find new ways to live with the illness, replacing old habits while maintaining the quality of life, continuing to live by priorities that are highly valued, despite one's illness. This means learning to deal with this new situation on the basis of what creates meaning.

One informant described how he still enjoys life and manages it with responsibility as follows: We eat delicious food. I think it's so good. You just have to learn to deal with it in a sensible way. The big thing for me is cooking at home because that gave me so much joy before I had diabetes.

Responsibility for supporting learning in a way that promotes health and well-being is also demonstrated in an eagerness to understand and interpret bodily signals, and to act on these. Knowledge of the body's blood sugar levels provides expanded scope for action. As another informant reflects on what happens with him, “I know it directly when I get too much sugar in me and I get tired.” This supports awareness that he must do something that requires concentration to ensure that his blood sugar is at a good level. Learning is supported by the person's own reflective responsibility which itself is promoted by curiosity and desire.

### Openness enabling support

Learning is also supported by openness about the illness and the requirements that follow the treatment. Openness plays an important role to get support from family, friends, and colleagues, as well as helping the person to reflect and find new thoughts and a new way to live. Work relationships can be supportive when colleagues are aware of the person's need for routines around breaks and meals; conversely, a lack of such understanding is not supportive. One informant described it in this way: “Before I let them know about my diabetes, the breaks and meals were not so regular, but now we have breaks at 8, 10 and 12, which is perfect.” Another informant said that colleagues were considerate about his needs: “We schedule the meetings to 14:00 to suit” (his need for regular meals).

Relatives and friends can both support and complicate the routines of everyday life. Informants described support in various ways, such as: “We agree that we should dine at 13:00, and then get others to say what they want, and we usually keep 4 h between meals.” Another informant said “My wife does it even harder than I do.” Concerning the difficulties of developing an understanding of the need for routines, one informant said “I want to eat at certain times, but my wife is not so firm,” adding:

The hardest thing is to do with my wife, she finds it hard to resist sweets; I have to nag her so she does not have it in view, but it is difficult, and then I cannot resist. It is not difficult to resist in the shop, but if it is in view at home or I'm offered, then it is difficult.

At a superficial level, clear rules can help to support learning “I follow the advice I got from the diabetes nurse and got good blood sugar,” but openness about how the body reacts provides support for learning at a deeper level. Experiments showing how food and exercise affect one's blood sugar can support learning if the person is open and reflects on the results. One informant described how he tests, observes, reflects, analyses, and reaches conclusions:

Quickly, after 2 hours, I could see what happened when I ate something. So I continued to test, and after 3 months, I had eaten my way through the entire range of foods. I knew that it was ok to eat salad, as it did not show up. Then I played around with it a bit more. Legumes worked pretty well, and later, I mixed legumes and salad; it was pretty okay, and I could eat my fill. Then I searched for good salads with beans, and just carried on.

While prescribed self-care methods can be experienced as superficially supporting learning to live with diabetes, the felt positive effects of lifestyle changes can promote deeper learning, making it easier to sustain those changes:

I understand that exercise is good, and I feel it is good, it's nice // if I have a cold or it's miserable weather and I don't go out, it is as if there is something missing // today, I have not been out and it feels strange // I have started something that will last, I hope.

In this way, routines and rules can help to support a change of behaviour; through openness, the person gets access to social support and the changed behaviour becomes normal and natural.

### Technology verifying bodily feelings

Technology that verifies knowledge and feelings in the body can also support learning to live with diabetes to promote health and well-being. For instance, blood glucose measurements support learning to understand the body's signals by training one's sensitivity to when the level is low or high. An informant who remembers when blood glucose meters were introduced describes it like this: “It was very exciting and very nice, as it enabled me to control my illness so that I knew I was at a good level.” Notes of blood sugar levels become the basis for dialogue and reflection on readings, which promotes learning about how various activities affect blood sugar levels. An unexpected result can activate reflection. What have I done now? What is different? One female informant described her realization that “negative thoughts and thinking everything is bad will not be good for your blood sugar level.”

Technology for measuring blood sugar levels assists understanding of the connection between food intake, activity, exercise, and mood or how one feels. This technology also makes it possible to monitor changes in blood sugar levels over time, which in itself supports learning “great to do a check during the night to see how blood sugar is when you are sleeping.”

To use the technology effectively, one must have goals to strive for; without knowledge of target values, the patient will be unable to experience how blood glucose measurements can support learning, and blood glucose measurements become worthless. As one informant described it:

I checked the blood sugar level a few times, starting in the morning, and saw the rise when I ate, but there were no big changes, somewhere around 5–6 and sometimes maybe 7. Maybe I should do it once a month, but it did not work for me anyway.

The ability to measure blood sugar levels is both attractive and frightening, and it can create ambivalent feelings. For most informants, it brings a positive feeling of safety and control, which helps to widen the boundaries. Some informants, however, expressed concern that the technology would take over to a point where they would not trust their body's signals; one woman put it like this:

When the diabetes nurse asked me if I wanted a blood glucose meter, I felt that I did not want a meter. I felt that it must not take over because I could end up pricking myself unnecessarily just to check. Therefore, I decided to wait as long as it works.

The same informant also said:

I do not think of the illness a lot. Sometimes, I think that in a way it would be nice to have the syringes so I could check and see what I can eat. Now, it will be more like, oh, what happened now? Why am I getting a bit dizzy? Maybe it will be like that later, too, I just don't know, but somehow it still feels like it would be more real.

Those affected cannot always connect the feeling in their body to their blood sugar level, as bodily feelings can be a signal of other bodily needs. Through reflection, however, the technique of measuring blood sugar, in combination with feelings in the body and food activities, can promote deeper learning.

In learning, to calculate the dose of insulin at mealtime, the technology for carbohydrate counting provides additional support. A blood glucose measurement verifies whether a dose is correct for the current situation, and the patient will remember and use this knowledge in similar subsequent situations. New technologies for monitoring blood sugar levels and dispensing medicine have made it possible to learn how to manage the illness, minimizing its impact on everyday life. As one informant described it:

I actually live like a healthy person, eat more sweets than average persons do (or some persons anyway). Actually, I think it's not good, but I have a good HbA1c, and I'm very careful to check myself.

The technology supports learning by providing more opportunities to adjust the treatment to the current situation, which means more freedom. Reflection plays an important role in making the patients feel safe, giving them courage to take on the new technology and to challenge their own understanding.

### A permissive climate providing for participation

Additional support for learning is found in a permissive environment where health care professionals involve the patient in designing their treatment, and where the patient feels involved in that planning. A female informant described it like this: “They cannot fool me; I have to agree to it myself and then be motivated, because I want to know what I need to do.” Participation—involving the patient in making decisions about what to do—is crucial. This participation in the caring relationship is also supported by experiments with subsequent reflective dialogue, in which theoretical and practical knowledge is combined to increase knowledge and responsibility. As one informant described it:

I have quite a lot of freedom and get a proposal. We try it, adjust the dosage in a certain way, and if it does not work, I can change units a little bit up and down. I think this feels pretty good. Because there is no one else who can solve it, I have to do it myself in order to live a reasonably normal and simple life.

To support participation and the creativity to dare to explore new possibilities, an open, equal, and trusting relationship with the diabetes team is important. In such a caring relationship, questioning is not perceived as threatening but as supportive of learning. On the other hand, professional support based on instructions and intimidation discourages learning. One informant described unsupportive information as “not emotional but strong facts that were given but difficult to follow,” and “when I got there and had high blood sugar levels, I was almost given a scolding.” When the diabetes team professionals hand over responsibility in keeping with the patient's increased knowledge, this creates a sense of security. Knowing that one always has the option of contacting the team increases self-confidence. As one informant said:

… that I could call if there is anything // during my pregnancy, I could call my doctor at any time, night or day. In this situation, you must have 100% backing, and it made you feel safe. I did not need to call, it was enough to know that I could, and that made me feel secure.

To be able to challenge their own understanding, it was also important that the patients felt they could trust the available health care contact person. The informant described a different sense of security when consulting staff with specialized knowledge of diabetes, as compared to the health care information service, where they felt the staff had only basic knowledge.

A climate of trust in the caring relationship demonstrates that some situations are more difficult to influence. This kind of trust was illustrated in the following terms: “The doctor agreed to a higher blood sugar level for some time, saying it was ok; we know what you have been through now.” Understanding supports patients to recognize the difficult situation, talk about it, and eventually turn it into something positive—“a bit like this entire life, really.” A permissive climate supports the learning experience, providing knowledge and increased understanding that one cannot always control everything that happens in life and so affects one's blood sugar level, no matter how hard you try. A permissive climate supports learning and opens the mind to the caring relationship, as well as to a humble approach to life, which seems to be important in living with diabetes.

### Exchanging experiences with others

Learning is supported by sharing experiences with others, such as professional carers, relatives, acquaintances, or other persons with diabetes. Experience exchange can take place in different ways, but it often starts a reflection process. Citing the example of a group meeting, one informant describes it as follows:

We sit in groups and talk, maybe with someone next to us // hearing and sharing a lot about practical things, what others have experienced and how they feel, or what they are experiencing now. There is much to learn from each other; we all react very differently, all of us are individuals.

For patients with newly diagnosed diabetes, experienced patients’ stories can be an awakening that supports the search for knowledge about the illness and how its development can be slowed. This was described by one informant in the following terms: “Hearing their stories was like getting a punch in the face.” The exchange of experiences can also prepare one for the challenges to come. In some cases, hearing about individual variations can increase understanding of one's own or others’ failures and how to overcome them. One informant describes it like this:

For me, it has worked, but I'm a little more humble now as to how others experience it. They come home in the evening and are going to cook something for the children, they should be full and it should be done fast, so it will be pasta. Then they cook something for themselves. It is really hard. I changed my mind after the course because I got to see a bit how others had it at home; it's not so simple, so I do not judge as I did in the beginning.

Experiences with others can enhance understanding and support learning when fears, thoughts, and feelings are put into words. The information a diabetes nurse tries to convey may be complemented by the exchange of experiences within the group. Information about insulin requirements can be daunting, but if it is described by persons using insulin without discomfort, that fear is reduced. Another form of support for learning is the exchange of experiences online. One informant reported such an exchange through an Internet chat room: “Some guys posted their Excel sheet, and I thought that I would do so too.” Accessing new channels means that knowledge can be supported by persons far beyond one's own network of contacts, increasing the possibility of finding knowledge that meets their personal needs.

Information presented in a way that suits an individual's way of assimilating new knowledge supports learning in a way that promotes health and well-being. Some participants found support by reading brochures, books, and the diabetes association's magazine, featuring research abstracts and the experiences of other patients. One woman told me how she read an article and reflected that “I have come to that conclusion myself,” confirming her own insight.

Others find the Internet to be a useful source of knowledge. As one informant described it:

On the Internet, you can search for many things. If there's something you have questions about, you can just google the question—a few words and you can see that there are many others who have thought and written about it before.

Learning is supported through the exchange of experience, and the reflection over it, and how others’ experiences can be understood in relation to one's own experiences. Knowledge and understanding increases, including the realization that it is not always easy to live with the illness, which creates humility about the task of learning to live with diabetes in a way that promote health and well-being.

## Discussion

The aim of this study was to describe the phenomenon of support for learning to live with diabetes to promote health and well-being, from the patient's perspective. This included self-responsibility, driven by reflection on experiences, curiosity, and a desire to understand and influence one's daily life. This together with openness about the illness and reflection supported by technology and a permissive climate promoted learning to live with diabetes. The study highlights that support for learning is three-dimensional: individual, professional, and social. Hupcey (1998) has defined support as social and professional. The third dimension that has been described in this study is the importance of the person's own responsibility to take charge in his/her own situation by being responsible, insightful, and reflective. Previous studies have shown that activities initiated and driven by patient needs can reinforce previous knowledge and support the ability to affect diabetes-related health, as measured by HbA1c (Tang, Funnell, Brown, & Kurlander, 2010). There is also evidence that patient-driven self-management support programmes can enhance diabetes management and self-care (Dam, Horst, Borne, Ryckman, & Crebolder, 2003) by increasing the frequency of healthy eating and monitoring of blood glucose (Durán et al., 2010; Tang et al., 2010).

In addressing the research question, the RLR approach (Dahlberg et al., 2008) was found useful and appropriate here, as participants in the present study were openhearted in communicating their many experiences of support for learning to live with diabetes. Because the aim of the study was to describe support for learning to live with diabetes, participants with both type 1 and type 2 diabetes have been included in the study. This is in line with Svedbo Engström, Leksell, Johansson, and Gudbjörnsdottir (2016). In relation to the phenomenon of the studies we do not believe that the type of diabetes is significant for the results. Effort has been made to get such a varied picture as possible of the phenomenon.

Throughout the study, the researchers sought to maintain an open position, and preconceptions were regularly reflected (Husserl, 1975). Bracketing these preconceptions to achieve a scientific and reflective position meant slowing down and remaining conscious of them at all times through critical questioning of the meanings in our results. All the authors participated in discussions to reach a deeper sense of the phenomenon and of the significance of the patients’ experiences. Although KJ and JL are diabetic nurses, the other authors are not and have been able to be more critically open. As the phenomenological approach allows for description of the richness and varied meaning of lifeworld phenomena, the meanings arrived at are abstractions that can ideally be generalized (Dahlberg et al., 2008), though with caution, as they are necessarily context-specific.

The roll of reflections in learning has previously been described in a different context by Bengtsson (1998), Berglund (2014), and Ekebergh (2007) following Heidegger (2008), Gadamer (1989), and Merleau-Ponty (1983, 1995). From a lifeworld perspective, reflection is seen as a process of understanding which is of crucial importance for learning (Ekebergh, 2007). The findings of the present study confirm that support promoting reflection plays a central role in learning among persons with diabetes. Reflection can, according to the results, be supported by the person himself/herself, for example, by analysing his/her actions in relation to blood glucose values; by social support, for example, by questions asked by relatives that start reflection; and by professional support, for example, by a permissive climate where the patient is allowed to reflect upon his/her failures.

Through reflection, new understanding is created, described by Gadamer (1989) as a horizon fusion of new experiences with previous understanding. *Reflection* refers to how an individual turns their attention inward to discover the self (Bengtsson, 1998). In this regard, it can be compared with contemplation and consideration. Gadamer (1996) argued that by adopting this critical distance from himself, a person can become reflectively aware of himself and his actions. In this study, reflection is found to support learning and, in particular, to be important for learning to live with diabetes. Wide support for the importance of reflection is confirmed here in the participants’ awareness of their responsibility for their own health process. According to Hörnsten, Jutterström, Audulv, and Lundman (2011), this emerges when the patient integrates the illness emotionally and existentially, learning through reflection and taking responsibility for understanding their own body (Johansson et al., 2015). A lived body that has been changed by illness will not be recognized and can be described as homeless (Gadamer, 2003; Svenaeus, 2011). Only by learning how the body works can the sense of insecurity and homelessness created by illness be mastered, in what earlier studies have described as “learning turning points” taking responsibility for one's actual situation and for what can and cannot be changed (Berglund, 2014).

The findings of the present study also show that reflection is supported by technology that can verify feelings and experiences relating to the body's expression of blood sugar. This can be explained by the variation theory described by Marton and Ming (2006). When the patient experiments, evaluates, and reflects on these results the result emerges as a variation, which supports learning by enhancing the patient's knowledge of how the changing body reacts and signals change. This is consistent with the results of other studies showing that technology can be used to supplement diabetes care, with positive impacts on HbA1c, self-management behaviours, and self-efficacy (Durán et al., 2010).

The present results highlight the need to know targets in order to reflect, evaluate, and reach conclusions. According to Berglund (2014), achieving objectives is important in realizing that you have learned something. Other studies have shown the importance of involving patients in setting their own goals (e.g., Wikblad, Leksell, & Smide, 2004). According to Hortensius et al. (2012), describing the importance of balance between achieving blood glucose targets and quality of life, blood sugar control can be both “friend” and “foe.” The present study shows how measuring blood sugar can make a patient feel safer, as it is sometimes difficult to interpret the lived body's signals. This is confirmed by Tan, Chen, Taylor, and Hegney (2012), who showed that some persons lack the necessary knowledge to interpret body signals in identifying and self-managing hypoglycaemia. Similarly, Kato, Cui, and Kato (2013) showed that structured self-monitoring of blood glucose increases knowledge of how the lived body reacts and awareness of the connection between food and blood sugar, leading to increased participation in treatment change (Polonsky et al., 2011).

The present results show that other people's stories can activate reflection and motivate change by awakening understanding of what has been done, one's current lifestyle, and its potential future consequences. The results also show that the people around you promote reflection and accountability in different ways, through professional or social support. A care relationship that fosters an open climate is important for creating reflection, trust, participation, and responsibility for treatment, described as an “inspiring” learning climate by Leksell, Sandberg, and Wikblad (2006). A climate of confidence is also important for articulation of the patient's fears, beliefs, and expectations (Janes, Titchener, Pere, Pere, & Senior, 2013), which have been shown to include fear of losing control and future complications and security with good control (Johansson et al., 2015). In line with the present findings, Frost, Garside, Cooper, and Britten (2014), show that professional support that senses the patient's level of maturity and gradually hands over responsibility can create a sense of safety and confidence. Through daring to talk about the fears associated with living with the disease in an open dialogue with the nurse emotional barriers for learning can be reduced.

The present results also confirm that support for learning is three-dimensional: individual, professional, and social. Relatives and friends contribute to social support, facilitating the integration of illness and the ability to create good habits, whereas the illness is complicated if unsuitable habits persist. It also became clear that other persons with diabetes can contribute with the sense of fellowship, recognition, experience sharing, and advice. Tang et al. (2010) described how a patient-directed intervention for lifelong management initiated a group dialogue about how to prevent and treat low blood glucose and so assisted problem solving. The results of the present study confirm that transparency about the illness and the exchange of experiences with others in the same situation supports accountability and the process of feeling “at home” again in the lived body. In other words, by understanding how one's body reacts in different situations and what it needs, it can be taken for granted. Again, this aligns with earlier evidence that the new can become the natural and regular (Johansson, Ekebergh, & Dahlberg, 2009), developing self-confidence and a new sense of coherence in life (Aujoulat, Marcolongo, Bonadiman, & Deccache, 2008).

The beneficial effect of group training on HbA1c is reported by the SBU (2009), again indicating the importance of social support. However, there is less clarity about how group training should be designed to promote existential learning and a sense of “home,” as suggested by the Steinbekk, Rygg, Lisulo, Rise, and Fretheim (2012) study which showed that the use of different measures of quality of life leave us with no clear picture. It remains a challenge for health care to promote interactions that create reflection and support responsibility, experimentation, and the search for knowledge, encompassing both biological markers and existential learning.

## Conclusion

Adopting a lifeworld perspective, the findings confirm that reflection is important in supporting learning to live with diabetes to promote health and well-being. Reflection is supported by a number of factors, including personal responsibility, transparency, technology, and exchange of experiences with others. For caregivers, the challenge is to create interactions in an open learning climate that will activate and promote reflection so that knowledge and experience are interwoven and integrated in personal learning.

For a caregiver, the challenge is to be creative and find new ways of working that meet patient care needs in a way that supports reflection.

For patients with diabetes, the challenge is to take responsibility for their own learning by being open, curious, and responsive in acquiring knowledge, learning from others experiences, and evaluating their own actions. To this end, patient associations, dialogue groups, workshops, and fiction chat clubs on the Internet can offer social support for individuals in their active efforts to learn.
